# Three separate acquisitions of *bla*_NDM-1_ in three different bacterial species from a single patient

**DOI:** 10.1007/s10096-023-04651-4

**Published:** 2023-09-09

**Authors:** L. F. Mataseje, J. Pitout, M. Croxen, M. R. Mulvey, T. C. Dingle

**Affiliations:** 1 National Microbiology laboratory, Winnipeg, MB Canada; 2 Alberta Precision Laboratories, Public Health Laboratory, 3030 Hospital Drive N.W, Calgary, AB T2N 4W4 Canada; 3 University of Calgary, Calgary, AB Canada; 4 University of Pretoria, Pretoria, Gauteng South Africa; 5 University of Alberta, Edmonton, AB Canada; 6 Li Ka Shing Institute of Virology, University of Alberta, Edmonton, AB Canada; 7 Women and Children’s Health Research Institute, University of Alberta, Edmonton, AB Canada

**Keywords:** Carbapenemases, Canada, New Delhi metallo-beta-lactamase, Pseudomonas, Acientobacter, Proteus

## Abstract

To investigate the acquisition and relatedness of New Delhi Metallo-beta-lactamase among multiple separate species from one patient. Five isolates from three species (*Pseudomonas aeruginosa*; Pa, *Acinetobacter baumannii*; Ab and *Proteus mirabilis*; Pm) suspected of harbouring a carbapenemase were investigated by phenotype (antimicrobial susceptibilities) and whole genome sequencing. Epidemiological data was collected on this patient. Three different carbapenemase genes were detected; *bla*_VIM-1_ (*Pa;* ST773), *bla*_OXA-23_ (*Ab*, ST499) and *bla*_NDM-1_ identified in all isolates. NDM regions were found chromosomally integrated in all isolates. Data showed no evidence of NDM-1 transfer within this patient suggesting the enzyme was acquired in three separate events.

**Supplementary Information:**

The online version contains supplementary material available at 10.1007/s10096-023-04651-4.

## Brief report

Gram-negative bacteria, most notably *Escherichia coli*, *Klebsiella pneumoniae*, *Pseudomonas aeruginosa*, and *Acinetobacter baumannii* were among the six most common antimicrobial-resistant (AMR) pathogens identified in a global 2019 report [1]. Carbapenem resistance due to carbapenemases is of concern as transfer between different bacterial species through mobile genetic elements, such as transposons and transmissible/conjugative plasmids are common [2].

Out-of-country hospitalization is an important risk factor for colonization or infection with carbapenemase-producing organisms (CPOs) [3, 4]. In Canada, patients with international travel one year prior were significantly more likely to have extensively drug-resistant carbapenemase-producing Enterobacteriales (XDR-CPE) than a non-XDR-CPE [5]. It is essential to rapidly identify patients colonized or infected by CPOs and place them on appropriate infection control precautions.

In June 2022, an elderly female with a lower urinary tract infection, hydronephrosis, and hyperglycemic crisis was admitted to the hospital. She was medevacked from a medical centre in Egypt where she was admitted in May 2022, with urosepsis secondary to a retained renal stone. She received ampicillin/sulbactam, ceftriaxone, meropenem, and moxifloxacin during her stay in Egypt. The patient was immediately placed on contact precautions. She did not receive antibiotics and no secondary spread was documented during her hospital stay in Canada.

Routine admission screening for antimicrobial-resistant organisms (hospitalization >24 hours outside of Canada within 6 months) was performed using rectal swabs which were sent to the clinical laboratory. Growth of three different Gram-negative bacteria was obtained on CHROMID® CARBA SMART Agar (bioMérieux Canada, Saint-Laurent, Quebec) and identified as *A. baumannii*, *P. aeruginosa*, and *Proteus mirabilis* respectively. These isolates were referred to the National Microbiology Laboratory (NML) in Winnipeg for carbapenemase testing. Subsequently, carbapenem-resistant *P. aeruginosa* (from urine) and *P. mirabilis* (from peritoneal fluid) were obtained within 7 days.

A total of five isolates from the three species harbouring a carbapenemase were sent for whole genome sequencing (WGS). Antimicrobial susceptibilities were determined (Sensititre, panel CANMSF1), which showed extensive drug resistance (XDR) in all isolates by Canadian recommendations [6] using CLSI M100, 32^nd^ edition interpretive criteria (Table 1). DNA was extracted using Qiagen DNeasy kits (Qiagen, Toronto, Canada) and sequenced on an Illumina NextSeq™ platform. MinION (Nanopore Technologies, Oxford, UK) sequencing was conducted using the rapid kit (SQK-RBK 004) on R9.4 flowcells and run on Guppy 6.3.7 using the super accurate base-calling model. De novo hybrid assemblies were done using Unicycler 0.4.7 [7]. Assembled sequence data was analyzed for Multi Locus Sequence Typing (MLST), antimicrobial resistance genes, and plasmid typing using the StarAMR tool (https://github.com/phac-nml/staramr).

**Table 1:** Phenotypic and genotypic data on all isolates collected from a single patient. MICs were interpreted using CLSI M100, 32^nd^ Edition. MICs did not differ between pairs of *P. mirabilis* or *P. aeruginosa* isolates

Site of isolation	N22-01347 (*A.baumannii*)		N22-02120 (*P.mirabilis*)		N22-01345 (*P.mirabilis*)		N22-01690 (*P.aeruginosa*)		N22-01752 (*P.aeruginosa*)	
	Rectal Swab		Peritoneal fluid		Rectal Swab		Rectal Swab		Urine	
Infection/Colonization	Colonization		Infection		Colonization		Colonization		Infection	
Sensititre CAN1MSF	MIC (mg/L)	Intrp	MIC (mg/L)	Intrp			MIC (mg/L)	Intrp		
Amikacin	>32	R	>32	R			>32	R		
Aztreonam	IR	-	>16	R			4	S		
Cefepime	>16	R	>16	R			>16	R		
Ceftazidime	>16	R	>16	R			>16	R		
Ceftazidime/avibactam	>16	NI	>16	R			>16	R		
Ceftolozone/tazobactam	>8	NI	>8	R			>8	R		
Ceftriaxone	>32	R	>32	R			IR	-		
Ciprofloxacin	>2	R	>2	R			>2	R		
Colistin	<=1	I	IR	-			2	I		
Doxycycline	<=4	S	>8	R			>8	NI		
Ertapenem	IR	-	>2	R			IR	-		
Gentamicin	>8	R	>8	R			>8	R		
Imipenem/relebactam	>8	NI	>8	NI			>8	R		
Levofloxacin	>4	R	>4	R			>4	R		
Meropenem	>16	R	>16	R			>16	R		
Meropenem/vaborbactam	>8	NI	>8	R			>8	NI		
Minocycline	<=4	S	>8	R			>8	NI		
Piperacillin/tazobactam	>64	R	64	R			>64	R		
Plazomicin	>8	NI	>8	R			>8	NI		
Tigecycline	<=0.5	NI	IR	-			IR	-		
Tobramycin	>8	R	>8	R			>8	R		
Trimethoprim/sulfamethoxazole	>4	R	>4	R			IR	-		
Data from WGS										
Sequence type	499		No scheme		No scheme		773		773	
AMR genes	aac(6’)-Ib-cr, aac(6’)-Ib3, aph(3”)-Ib, aph(3’)-VI, aph(3’)-VIa, aph(6)-Id, armA, ARR-3, blaADC-25, blaGES-35, blaNDM-1, blaOXA-23, blaOXA-95, cmlA1, dfrA7, mph(E), msr(E), qacE, sul1, sul2		aac(6’)-Ib, aac(6’)-Ib-cr, aadA1, ant(2”)-Ia, aph(3’)-Ia, aph(3’)-VI, armA, blaNDM-1, blaVEB-6, cat, dfrA1, dfrA5, mph(A), mph(E), msr(E), qacE, qnrA1, qnrS1, sul1, tet(A), tet(J)		aac(6’)-Ib, aac(6’)-Ib-cr, aadA1, ant(2”)-Ia, aph(3’)-VI, armA, blaNDM-1, blaVEB-6, cat, dfrA1, dfrA5, mph(E), msr(E), qacE, qnrA1, qnrS1, sul1, tet(A), tet(J)		aadA11, aph(3’)-IIb, blaNDM-1, blaOXA-395, blaPAO, catB7, fosA, qacE, qnrVC1, rmtB, sul1, sul1, tet(G)		aac(6’)-Il, aadA11, aadA2b, ant(2”)-Ia, aph(3’)-IIb, aph(3’)-VI, blaNDM-1, blaOXA-392, blaOXA-395, blaPAO, blaVIM-1, catB7, fosA, qacE, qnrVC1, qnrVC1, rmtB, sul1, tet(G)	
Plasmid Finder data	none detected		none detected		none detected		none detected		none detected	

*Intrp* interpretation, *S* susceptible, *I* intermediate, *R* resistant, *IR* intrinsically resistant, *NI* no interpretation, *IR* intrinsic resistance

Overall, three carbapenemases were detected; *bla*_VIM-1_ (in one of two *P. aeruginosa*), *bla*_OXA-23_ (*A. baumannii,* two copies), and *bla*_NDM-1_ (*P. aeruginosa, A. baumannii, P. mirabilis*). Interestingly, *bla*_GES-35_ was identified from the *A. baumannii* isolate. The *bla*_GES-35_ sequence was available on NCBI and identified from a *K. pneumoniae* and an *A. baumannii* isolate (accession WP_111273848, AWN81339). A report from Egypt also mentions the identification of this variant [8]. There were no mutations in the Omega Loop (guanine was present at amino acid position 170) known to be characteristic of carbapenemase activity in *bla*_GES_-variants [9]. It most closely resembles *bla*_GES-22_ a known β-lactamase [10] and differs by one amino acid within a region not shown to contribute to carbapenemase activity. The *A. baumannii* belonged to ST499^Pas^, which has recently been described as the emerging dominant non-clonal complex 2 carbapenem-resistant *A. baumannii* lineage in US hospitals [11]. The isolate in this study harboured two copies of *bla*_OXA-23_, one on a plasmid and one on the chromosome. Though not an uncommon occurrence, one study showed *bla*_OXA-23_ co-occurrence on chromosomes and plasmids altered bacterial phenotypes that are important for bacterial fitness such as better competitive growth, serum tolerance, and biofilm formation capacity [12]. Additionally, this isolate harboured both *bla*_OXA-23_ and *bla*_GES-35_ on an 80Kb plasmid (pN22-01347_B) belonging to rep group RP-T1 [13]. Using PLASDB (https://ccb-microbe.cs.uni-saarland.de/plsdb/) it was found that plasmids from USA [14] and Germany contained genetic content highly similar to pN22-01347_B, with the exception of a 2.8Kb region harbouring *bla*_OXA-23_ (accession numbers CP008707, CP087311; Figure S1a). This 2.8Kb region was associated with a partial Tn*2007* composite transposon previously shown to be associated with *bla*_OXA-23_ dissemination [15, 16]. Like previous reports [11, 14] pN22-01347_B contained a resistance island characterized by flanking 5-bp direct repeats of a 439-bp miniature inverted-repeat transposable element (MITE)-like sequence. This 6 Kb island was inserted between an integrase and the transposition protein TniB and included the resistance genes *aac(6’)-lb3, blaGES-35, aph(3’)-Vla, drfA7, qacE-delta, and sul1*. The presence of these resistance genes in a putatively mobile genetic element could greatly enhance resistance spread to other bacteria.

Both *P. aeruginosa* isolates belonged to ST773, serogroup O11. Core single nucleotide variant (SNV) analysis was conducted using the SNVPhly workflow [17], where 5 SNV differences (representing 99% of the genome) were observed between the core genome of the two isolates. Interestingly, *bla*_VIM-1_ was only found in one isolate (N22-01752) on a 450Kb circular plasmid (pN22-01752_A). When querying pN22-01752_A against the PLASDB similar plasmids were found belonging to IncP-2-type megaplasmids (ranging ~350–550Kb) isolated from China (NZ_CP073083) and Poland (NZ_MT732183, NZ_MT732197) among other countries (Fig. S1b). These are known to be associated with metallo-beta-lactamase-producing *P. aeruginosa* and have been identified in clinical and environmental isolates worldwide [18, 19]. Previous work on these IncP-2-type plasmids has shown its contribution in driving the dissemination of multi-drug resistance in *P. aeruginosa* [18, 19] Indeed, pN22-01752_A harboured the AMR genes; *aac(6’)-ll, aadA11 and A2b, ant(2”)-la, aph(3’)-VI, bla*_VIM-1_*, bla*_OXA-392-like_, *qacE, qnrVC1* and *sul1*. This plasmid was not present in the second *P. aeruginosa* isolate.

When investigating *P. mirabilis*, no plasmids were observed and only 2 SNVs were observed in the core genome (representing 99% of the genome) between the two isolates. Additionally, one isolate (N22-02120) contained two separate regions (6.2Kb and 6.4Kb) each flanked by IS*26* and containing additional resistance genes (*qacE*-delta, *sul1*, *mph*(A), *aph(3’)-la*) not present in the other *P.mirablis*. Important to the pathogenesis of *P. mirabilis* is the presence of several virulence factors that aid in adhesion and contribute to biofilm formation (MR/P, PMF, and UCA) which results in severe urinary tract infection [20]. Additional virulence factors such as phosphate transport (Pst), proteobactin (Pbt), and nonribosomal synthetase (NRPS) have been described in *P. mirabilis* [21]. Using the Virulence factors database (VFDB) (http://www.mgc.ac.cn/cgi-bin/VFs/v5/main.cgi) we identified previously described virulence genes [20, 21] including *mrpA-J*, UCA, *hpmA/B*, *zapA*, *pmfA,C-E*, *pbtA,B,D-I*, *nrpA,B,G,R-T*.

**Fig. 1:**
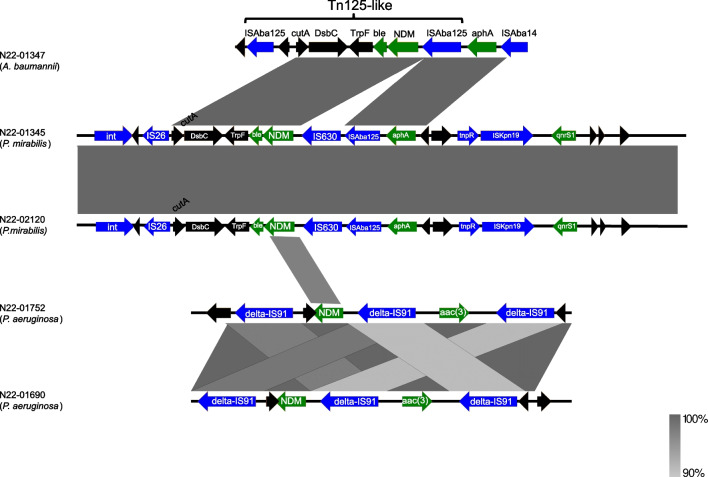
Schematic representation of NDM regions aligned between the three bacterial species in this study. Green represents resistance genes, blue represents mobile genetic elements and black are all other CDSs

NDM regions were found chromosomally integrated in all isolates and were compared as shown in Fig. 1. Data showed the presence of a partial Tn*125* in the *A. baumannii* isolate, which contained flanking copies of IS*Aba125* in addition to *cutA, dsbC, trpF,* and *ble.* Tn*125* has been well described in *A. baumannii* and linked to the dissemination of *bla*_NDM-1_ in this species [22]. The *P. mirabilis bla*_NDM-1_ region differed by the insertion of IS*630* between IS*Aba125* and *bla*_NDM-1_ as well as the presence of IS*26* adjacent to *cutA* (Fig. 1). This region and the surrounding 25 Kb in the *P. mirabilis* isolates were similar to several *K. pneumoniae* NDM plasmids described in NCBI (accession numbers CP050380, ON081621, MW911671), possibly suggesting a partial plasmid integration event into the *P. mirabilis* genome. Unfortunately, we could not identify specific genetic artifacts of where in the chromosome this occurred. The *P. aeruginosa* isolates had no similarity in surrounding NDM regions to either the *P. mirabilis* or the *A. baumannii*. Here *bla*_NDM-1_ was found inserted between two copies of a truncated IS*91*-like sequence. Similar to reports of NDM-1 harbouring *P. aeruginosa* ST773 [23] and ST234 [24] here, we observed *bla*_NDM-1_ on a putative integrative conjugative element (ICE) with a type four secretion system. The ICE was 116997bp flanked by *attL* and *attR* 23bp direct repeats inserted into tRNA. Overall, the NDM analysis in the various species suggested the patient acquired bacteria harbouring *bla*_NDM-1_ in three separate events.

Although the occurrence of multiple carbapenemases within a single patient has been commonly reported [25–27], it is important to highlight this case for several reasons. First, the *A. baumannii* isolate was shown to be an emerging clonal lineage (ST499) and contained duplicated copies of *bla*_OXA-23_, which has been previously shown to provide advantages to the fitness of the isolate [11]. Second, this patient also harboured a *P. aeruginosa* isolate that contained a previously described multi-resistant plasmid known to contribute to the dissemination of resistance genes in this species. Though the goal of this study was to investigate the relationship of *bla*_NDM-1_ across these isolates we revealed a complex collection of XDR pathogenic bacterial species that have the potential to rapidly spread multi-drug resistance within a hospital site.

### Supplementary information

Fig. 2

ESM 1

High resolution image

ESM 2

High resolution image

## Data Availability

Sequence data was uploaded to NCBI (BioProject PRJNA948358).
