# Supplementary figures and images for: Three separate acquisitions of blaNDM-1 in three different bacterial species from a single patient
Source: Eur J Clin Microbiol Infect Dis. 2023 Sep 9;42(10):1275–80. doi: 10.1007/s10096-023-04651-4 (PMC10511597; doi:10.1007/s10096-023-04651-4)

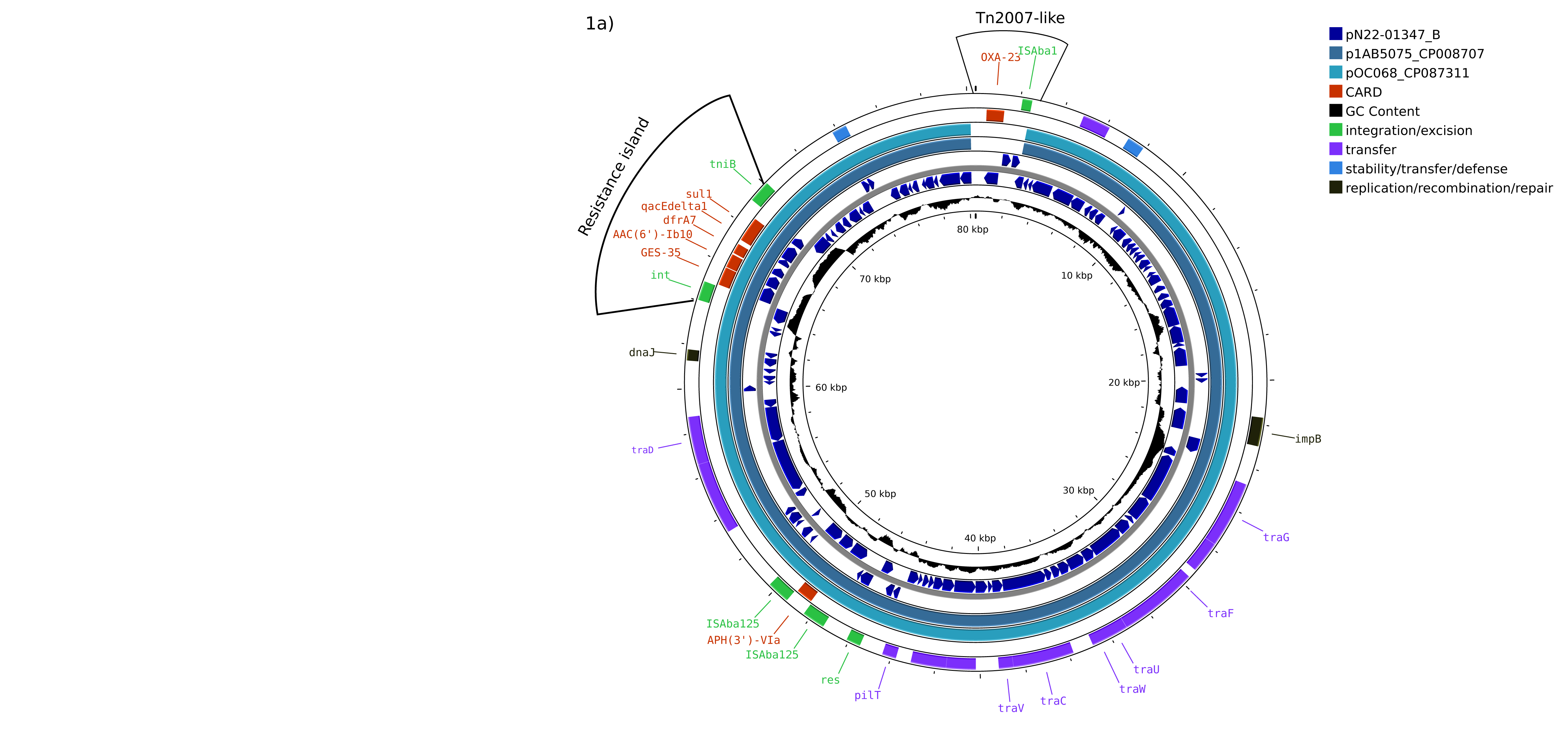

Supplement: Supplementary file 1 — ESM 1 [file 10096_2023_4651_Fig2_ESM.png]

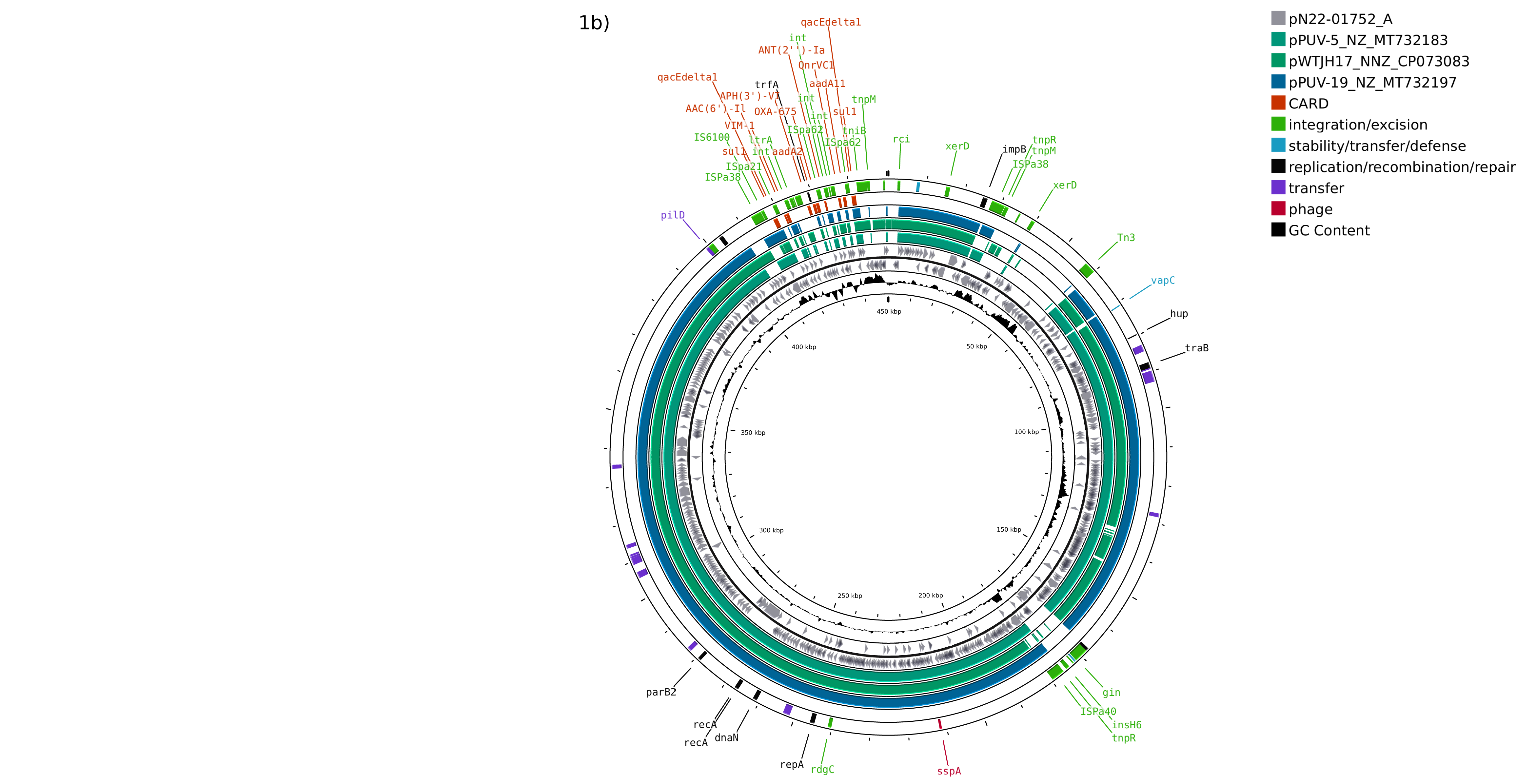

Supplement: Supplementary file 3 — ESM 2 [file 10096_2023_4651_Fig3_ESM.png]
